# The house cricket (Acheta domesticus) as a scalable invertebrate model for gerotherapeutic testing

**DOI:** 10.1093/geroni/igaf122.533

**Published:** 2025-12-31

**Authors:** Gerald Liao, Jenna Klug, Swastik Singh, Sherwin Dai, Elizabeth Bae, Warren Ladiges

**Affiliations:** University of Washington, Seattle, Washington, United States; University of Washington, Seattle, Washington, United States; University of Washington, Seattle, Washington, United States; University of Washington, Seattle, Washington, United States; University of Washington, Seattle, Washington, United States; University of Washington, Seattle, Washington, United States

## Abstract

The house cricket (Acheta domesticus) is a promising preclinical geroscience model due to its short lifespan, low maintenance, age-associated functional decline, and responsiveness to geroprotective drugs. Continuous dosing with rapamycin, acarbose, and phenylbutyrate extends lifespan; whether intermittent dosing offers similar benefits remains unknown. We tested 274 sex-matched crickets given 2-week intermittent dosing of each drug starting at mid-age (8-weeks), followed by behavioral testing at 10-weeks (geriatric stage). Assays included Y-maze olfactory discrimination, open-field exploration, and treadmill performance. Locomotor gaits were identified by velocity-based K-means clustering (silhouette > 0.5). A subset was monitored for post-treatment survival using Kaplan-Meier analysis. Olfactory preference was preserved by all drugs (d = -1.82 to -1.28, P’s < 0.01), with strongest effects in rapamycin-treated individuals. Rapamycin-treated males matched or exceeded juvenile locomotor activity; phenylbutyrate reduced male activity (d = 1.49, P = 0.049) and acarbose increased walking-to-running ratios (d = -0.75, P = 0.029). Rapamycin increased central exploration and freezing (d = -1.55, P < 0.0001), while acarbose and phenylbutyrate increased peripheral freezing (d = -0.76, P = 0.02). Rapamycin and phenylbutyrate extended maximum running time (d = -2.30 to -1.32, P < 0.0001), with sex-specific jumping gains in rapamycin-treated females and acarbose-treated males. Post-treatment lifespan was prolonged by rapamycin (HR = 0.42, P = 0.0004) and reduced by acarbose in females (HR = 2.92 to 3.03, P’s < 0.05). Intermittent rapamycin preserved survival, cognition, and locomotion, while acarbose and phenylbutyrate produced selective benefits, supporting A. domesticus as a scalable model for geroprotective drug discovery.

